# Gastro- or Duodenojejunostomy Leaks After Pancreatoduodenectomy: Single Center Experience and Narrative Literature Review

**DOI:** 10.1007/s11605-021-05058-2

**Published:** 2021-06-15

**Authors:** Knut Jørgen Labori, Tore Tholfsen, Sheraz Yaqub, Kristoffer Lassen, Dyre Kleive, Anne Waage

**Affiliations:** 1 Department of Hepato-Pancreato-Biliary Surgery, Oslo University Hospital, Rikshospitalet, Oslo, Norway; 2 Institute of Clinical Medicine, University of Oslo, Oslo, Norway; 3 Institute of Clinical Medicine, Arctic University of Norway, Tromsø, Norway

**Keywords:** : Pancreatoduodenectomy, Gastroenterostomy, Duodenoenterostomy, Leakage

## Abstract

**Background and Methods:**

Gastro- or duodenojejunostomy leaks after pancreatoduodenectomy is rare. This study aims to analyze the incidence, management, and outcome of gastro- or duodenojejunostomy leaks after pancreatoduodenectomy based on a single center experience from 2004 to 2020 with a narrative literature review.

**Results:**

Of a total of 1494 pancreatoduodenectomies, eight patients with gastrojejunostomy (n=1) or duodenojejunostomy (n=7) leak were identified from the institutional pancreatic database. All leaks were treated operatively. In two patients dismantling of the duodenojejunostomy, distal gastrectomy, and closure of the pyloric and jejunal side, a percutaneous endoscopic gastrostomy and a feeding jejunostomy ultimately had to be performed after an unsuccessful attempt of gastrojejunostomy and suture of the duodenojejunostomy, respectively. The literature search revealed three more studies specifically addressing this complication after pancreatoduodenectomy (36 patients of a total of 4739 pancreatoduodenectomies). Based on an analysis of the current study and the literature review, the overall incidence of gastro- or duodenojejunostomy leaks after pancreatoduodenectomy was 0.71 % (44/6233 pancreatoduodenectomies). The occurrence of a gastro- or duodenojejunostomy leak was associated with a concomitant postoperative pancreatic fistula in 50 % of the cases, an increased length of hospital stay, and a mortality rate of 15.9 %. Surgical treatment was performed in 84 % of the cases.

**Conclusion:**

Gastro- or duodenojejunostomy leak is a rare complication after pancreatoduodenectomy. Prompt diagnosis and early repair is important. In most cases, a surgical intervention is necessary for a good outcome. Under salvage conditions, a bailout strategy may be to temporarily dismantle the gastro- or duodenojejunal anastomosis.

## Introduction

A gastrojejunostomy (GJ) is part of the reconstruction in many surgical procedures. Classic pancreatoduodenectomy (cPD) with distal gastrectomy and GJ and pylorus-preserving pancreatoduodenectomy (PPPD) with duodenojejunostomy (DJ) are considered equally effective for the treatment of pancreatic and periampullary tumors [1, 2]. Anastomotic leakage of the GJ or DJ after PD is rare and can sometimes be severe and difficult to manage. There are limited publications focusing on this potentially serious complication after PD [1, 3–5]. This study aims to analyze the incidence, management, and outcome of GJ or DJ leaks after PD based on single center experience and a literature review.

## Material and Methods

From 2004 to 2020, eight patients with GJ or DJ leak were identified from the institutional pancreatic database of 1494 PDs. Patients were scored as having GJ or DJ leakage when a defect at this anastomosis was encountered at reoperation or when there was sufficient radiologic evidence of leakage [4]. PD was performed as previously described [6, 7]. From 2004 to 2011, pancreatic surgery in Oslo was performed at two different hospitals: Ullevål University Hospital and Rikshospitalet. In October 2011, the departments merged into the high-volume HPB surgical center it is today, with all procedures being performed at Rikshospitalet. From 2004 to 2011, Ullevål University Hospital had PPPD as standard procedure, whereas cPD procedure was preferred at Rikshospitalet. PPPD has been the standard approach since 2012. However, a distal gastrectomy is performed when there is a question of ischemia or tumor involvement of the proximal duodenum. Antecolic GJ or DJ was performed approximately 40 cm distal to the hepaticojejunostomy by single running continuous monofilament 4-0 suture. A surgical drain was placed in all patients. The nasogastric tube was removed immediately after skin closure and before endotracheal extubation [7]. There were no dietary restrictions after surgery, but patients were encouraged to begin carefully and increase intake according to tolerance over postoperative day (POD) 1–4. As a general rule, well-nourished patients not achieving adequate energy/protein requirement by oral intake within 5 days after the surgery received artificial nutritional support. Malnourished patients and those who developed severe postoperative complications early after operation received early supplementary artificial nutrition.

Clinical presentation, radiologic findings, treatment, and outcome of patients with GJ or DJ leakage were analyzed. Comprehensive complication index (CCI) and alternative fistula risk score (aFRS) were measured by means of the online tools (https://www.assessurgery.com/about_cci-calcula- tor/) (https://www.evidencio.com/models/show/621). Postoperative pancreatic fistulas (POPF), postpancreatectomy hemorrhage, and bile leaks were defined and graded according to international definitions [8–10]. The hospital review board approved the study (19/04710) according to the general guidelines provided by the regional ethics committee. Continuous variables are presented as a median (range).

A PubMed/MEDLINE literature search was conducted using the search terms alone and in combination of “pancreatoduodenectomy” OR “pancreaticoduodenectomy” AND “gastrojejunostomy” OR “duodenojejunostomy” OR “gastroenterostomy” OR “duodenoenterostomy” AND “leakage” OR “fistulas.” The search period ended as of 31 December 2020. Reference lists of all included papers and related articles were screened manually to identify missed but relevant studies. The final inclusion of papers to cite and reference was made at the discretion of the authors.

## Results

Eight patients (male n=5, median age 63 years) with a GJ or DJ leak were identified in the series of 1494 PDs, resulting in an incidence of 0.54 %. Details on surgical procedure, histological diagnosis, clinical presentation, and the postoperative course are presented in Table 1. Seven patients underwent PPPD, and in one patient, a cPD was performed. Six patients had a concomitant POPF grade C and one patient a POPF grade B. Median CCI was 81.9, and length of hospital stay was 84 days. Ninety-day mortality was zero. One patient died in hospital 18 months after a PPPD from complications related to the initial DJ leak diagnosed on POD 6. He had undergone extensive radiation therapy to the abdomen 4 decades previously.

**Table 1:** Surgical procedure, clinical presentation, and postoperative outcome in patients developing gastro- or duodenojejunostomy leak after pancreatoduodenectomy

Patient	Index operation	Histology	Diagnosis POD (days)	Clinical presentation	Diagnosis	Alternative FRS	POPF grade	PPH grade	Bile leak grade	CCI score	Hospital discharge (days)	Readmission
1	cPD	Distal cholangiocarcinoma	34	Intraabdominal abscess	CT	15.6	3	0	0	90.6	202	No
2	PPPD + left nephrectomy	Duodenal cancer	5	Air + intestinal content on drains	Clinical	16.8	3	1	0	94	84	Yes
3	PPPD	Chronic pancreatitis	13	Respiratory failure + wound dehiscence	Clinical	7	0	0	3	81	83	Yes
4	PPPD	Ampullary cancer	8	Intestinal content on drains	CT	19.1	3	0	0	65.2	31	No
5	PPPD	Ampullary cancer	6	Bile content on drains	CT	16.8	3	0		100	563	Yes
6	PPPD	Ampullary neuroendocrine carcinoma	4	Bile content on drains	CT	43.4	3	0	0	82.8	124	Yes
7	PPPD	Benign ampullary tumor	7	Peritonitis	CT	13.6	3	1	0	79.6	49	No
8	PPPD + portal vein resection + right hemicolectomy	Pancreatic cancer	8	PPH + peritonitis	CT	3.5	2	1	0	67.6	19	No
Overall			8			16.2				81.9	84	

*CCI* comprehensive complication index, *CT* computer tomography, *cPD* classic pancreatoduodenectomy, *FRS* fistula risk score, *POPF* postoperative pancreatic fistula, *POD* postoperative day, *PPH* postpancreatectomy hemorrhage, *PPPD* pylorus-preserving pancreatoduodenectomy

The leaks presented as intestinal fluid, bile, or air on the surgical drains in four patients. In the remaining four patients, the leak presented as intraabdominal abscess, peritonitis, wound dehiscence/respiratory failure, or postpancreatectomy hemorrhage, respectively. The leaks were diagnosed on median POD 8 (range 4–34). All cases underwent surgery as definitive treatment of the leak. In six patients, there was a high grade of clinical and radiological suspicion of a GJ or DJ leak before relaparotomy. In one patient, an initial conservative approach with percutaneous drains was chosen, but the patient finally underwent a relaparotomy eight days after the diagnosis of a DJ leak.

**Fig. 1:**
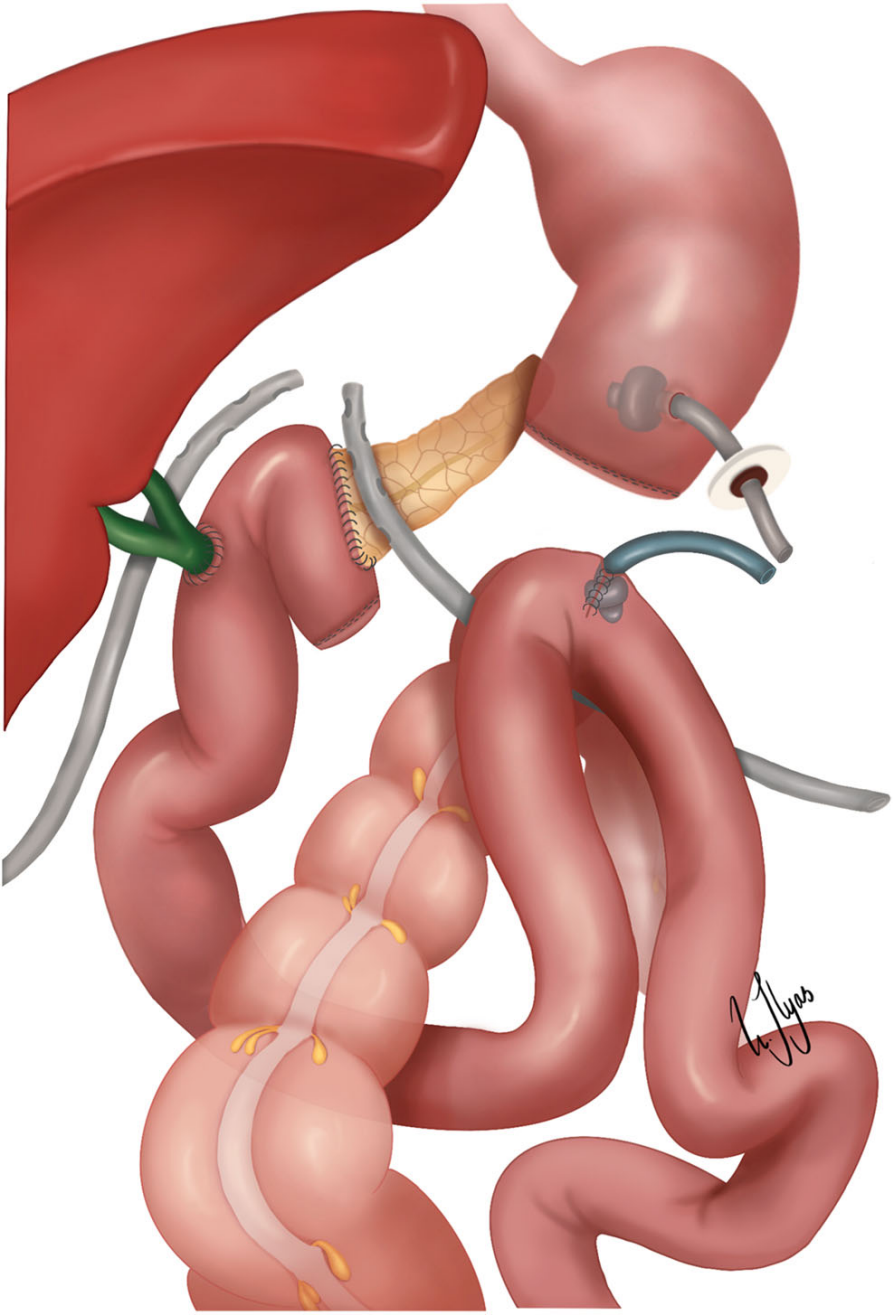
Bailout strategy used under salvage conditions in two cases of duodenojejunostomy leaks after pancreatoduodenectomy. After dismantling of the gastro- or duodenojejunostomy, distal gastrectomy, and closure of the pyloric and jejunal side, a percutaneous gastrostomy (PEG, Mic Key®) was applied with aid of a gastroscope, and the gastric serosal wall around the catheter was secured to the anterior abdominal wall. Enteral access for nutrition was fashioned with a 16 Fr Foley catheter through the closed jejunal defect in the first patient and 5 cm distal of the jejunal defect in the second patient. The jejunal serosal wall around the enteral catheter was firmly sutured to the anterior abdominal wall in a wide area around the catheter. Drains were placed for drainage of a concomitant pancreatic fistula. After healing of the concomitant fistulas, resolution of inflammatory changes, and restitution of nutrition, a gastrojejunostomy was safely performed after about 6 months

Table 2 displays the surgical management of the eight patients. Suture of the DJ defect was performed successfully in two patients. Following this procedure, one of these patients had two reoperations: first a completion pancreatectomy followed by yet another suture of the DJ defect. In one patient, a glue injection into the pancreatic duct was performed due to POPF grade C. Following this procedure, the patient had two reoperations: first a suture of the GJ defect followed by a redo GJ with Roux-en-Y reconstruction. Two patients had a reoperation with redo GJ, and one of the patients also underwent a redo hepaticojejunostomy; both patients had a successful outcome. Three patients had a particularly complicated course. One patient eventually died after 18 months of hospitalization due to complications related to the DJ leak, POPF, enteral leak from the blind end of the pancreatobiliary jejunal limb, intestinal obstruction, and massive intestinal adhesions. In two patients, dismantling of the GJ and DJ was performed after an unsuccessful GJ without Roux-en-Y reconstruction and suture of the DJ defect, respectively (Table 2, Fig. 1). After the dismantling procedure, a percutaneous endoscopic gastrostomy (PEG, Mic Key®) catheter was inserted for drainage of the stomach, in addition to broad drainage of the concomitant POPF. Both patients had a 16 Fr Foley catheter securely placed into the efferent jejunal limb for enteral nutrition (Fig. 1). These two patients finally recovered, and a reoperation with a GJ with and without Roux-en-Y reconstruction was successfully performed 139 and 176 days after the dismantling procedure, respectively.

**Table 2:** Surgical treatment of patients developing gastro- or duodenojejunostomy leak after pancreatoduodenectomy

Patient	DJ+GJ fistula treatment*
1	1. Pancreatic duct occlusion
	2. Suture of GJ
	3. Redo GJ with Roux-en-Y reconstruction
2	1. Distal gastrectomy and GJ
	2. Pancreatic duct occlusion, dismantling of GJ
	3. Percutaneous endoscopic gastrostomy and Foley catheter 16 Fr jejunostomy
	4. GJ without Roux-en-Y reconstruction 196 days after PPPD, 176 days after the dismantling procedure
3	Distal gastrectomy, GJ, and redo HJ
4	Distal gastrectomy and GJ with Roux-en-Y reconstruction
5	Initially conservative 1. Distal gastrectomy and GJ with Roux-en-Y reconstruction, suture of PJ
	2. Drainage
	3. Foley catheter 16 Fr gastrostomy
	4. Drainage
	5. Foley catheter 16 Fr jejunostomy
	6. Stenting of GJ, enteroraffia
	7. Enteroraffia
	8. Gastroraffia, enteroraffia, PTBD
	9. Redo GJ, entero-entero anastomosis
	10. Foley catheter 12 Fr jejunostomy
	11. Gastroraffia, enteroraffia, entero-entero anastomosis, colo-colo anastomosis
	12. ERP with pancreatic duct occlusion
	13. Gastrectomy, redo HJ, colostomy, colectomy, redo GJ with Roux-en-Y reconstruction
6	1. Suture of DJ
	2. Drainage
	3. a) Dismantling of DJ, distal gastrectomy, percutaneous endoscopic gastrostomy, jejunoraffia of jejunal defect after DJ, b) Second look following day: drainage, Foley catheter 16 Fr jejunostomy (through leak in jejunoraffia)
	4. Drainage
	5. GJ with Roux-en-Y reconstruction 160 days after PPPD, 139 days after the dismantling procedure
7	1. Suture of DJ
	2. Completion pancreatectomy
	3. Suture of DJ
8	Suture of DJ, resection of ileocolic anastomosis, end ileostomy, suture of PJ, hemostasis

*Numbers indicating subsequent relaparotomies with technical solutions

*DJ* duodenojejunostomy, *ERP* endoscopic retrograde pancreatography, *GJ* gastrojejunostomy, *HJ* hepaticojejunostomy, *PJ* pancreaticojejunostomy, *PPPD* pylorus-preserving pancreatoduodenectomy, *PTBD* percutaneous transhepatic biliary drainage

The literature search revealed three studies including 36 patients developing a GJ/DJ leak after a total of 4739 PDs (Table 3). Including the current case series, the overall incidence of GJ/DJ leak after PD was 0.71 %. GJ/DJ leaks were associated with concomitant POPF in 50 % of the cases, increased length of hospital stay, and a mortality rate of 15.9 %.

**Table 3:** Published series specifically addressing gastro- or duodenojejunostomy leak after pancreatoduodenectomy

Author/year	Number of patients	DJ or GJ	Median time between index operation and diagnosis of leakage (days, range)	Concomitant POPF n (%)	Treatment	Median length of stay (days)	Mortality n (%)
Winter 2008	13/3029=0.43 %	DJ 11 GJ 2	10 days (6–20)	5 (38)	Surgery 12 - Distal gastrectomy and GJ 10 - DJ revision 2 Percutaneous drain 1	35	4 (30.7)
Eshuis 2014	12/1036=1.16 %	DJ 11 GJ 1	8 days (2–23)	5 (42)	Surgery 7 - Distal gastrectomy + GJ: - with Roux-en-Y reconstruction 3 - without Roux-en-Y reconstruction 2 - Redo DJ with Roux-en-Y reconstruction 2 Percutaneous drain 4 None 1	41	1 (8.3)
Mazza 2019	11/674=1.63 %	DJ 9 GJ 2	13 days (5–45)	5 (45)	Surgery 10 - Distal gastrectomy + GJ with Roux-en-Y reconstruction 6 - Raffia 4 Percutaneous drain 1	38	1 (9.1)
Current series 2021	8/1494=0.54 %	DJ 7 GJ 1	7.5 days (4–34)	7 (88)	Surgery 8 - Distal gastrectomy + GJ with Roux-en-Y reconstruction 3 - Distal gastrectomy + redo GJ 1 - DJ/GJ revision 2 - Dismantling of DJ, later distal gastrectomy + GJ 2	83.5	1 (12.5)
Overall	44/6233=0.71 %	DJ 38 GJ 6	-	22 (50)	Surgery 37 Percutaneous drain 6 None 1	-	7/44=15.9 %

*POPF* postoperative pancreatic fistula

## Discussion

The current case series and literature review found an incidence of GJ/DJ leak after PD of 0.71 %. The occurrence of a GJ/DJ leak was associated with a concomitant POPF in 50 % of the cases, an increased length of hospital stay, and a mortality rate of 15.9 %. Surgical treatment was performed in 84 % of the cases, and all studies emphasized the importance of prompt diagnosis and surgery as the preferred treatment approach.

Three studies evaluated risk factors for GJ/DJ leak after PD [3–5]. Winter et al. found that perioperative risk factors included a preoperative blood urea nitrogen-to-creatinine ratio >20 (odds ratio=6; p=0.01) and intraoperative blood loss ≥1 L (odds ratio=6; p=0.03) in a multivariate model [3]. In a case-control study, Mazza et al. found that cases developing GJ/DJ leak showed lower preoperative serum hemoglobin (p=0.021) and increased preoperative radiotherapy (p=0.037) [5]. Moreover, these patients experienced a more demanding intraoperative course including an increased estimated blood loss (median 600 vs. 400 mL, p= 0.002), a higher rate of blood transfusion ( 31% vs. 8%; p=0.047), and a longer operative time (median 360 vs. 318 min; p=0.038). Eshuis et al. found that an additional organ resection during the index procedure was significantly more frequently performed and that the operation time was significantly longer (366 vs 301 min; p =0.001) in patients with GJ leakage [4]. Because of the low number of events, multivariate analysis for the identification of possible risk factors for DJ/GJ leakage was not performed in the studies by Mazza and Eshuis. Although based on small study samples, patients developing GJ/DJ leak after PD seems to have undergone a more demanding PD with longer operative time and increased intraoperative blood loss.

A difference between DJ and GJ in the occurrence of a leak has not been reported. Huttner et al. performed a Cochrane review to compare the effectiveness of cPD and PPPD and identified eight randomized clinical trials with a total of 512 participants [2]. The review revealed no relevant differences in mortality, morbidity, and survival between the two operations, and no analysis or comments on the GJ/DJ leak rate was made in that review. A search of the individual eight papers revealed only five reported cases of GJ/DJ leak, in the largest series by Tran et al. of 170 consecutive patients (n=2) and in a smaller series of 27 patients by Srinarmwong (n=3) [1, 11]. Moreover, in a systematic review and meta-analysis, Hajibandeh et al. found no significant difference in the incidence of anastomotic leak between stapled anastomosis (SA) versus hand-sewn anastomosis of GJ or DJ in PD [12].

In the current study, all patients underwent a relaparotomy for definitive treatment of the leak, although a conservative approach was initially attempted in one patient. Eshuis et al. reported four cases successfully treated with percutaneous drainage alone, whereas Winter and Mazza treated one patient each with percutaneous drainage alone [3–5]. Overall, surgery was the treatment of choice in 84 % of the patients (Table 3), and in all studies, the authors emphasized the importance of prompt diagnosis and operative intervention for a good outcome. Review of the literature revealed only one successful attempt with endoscopic treatment for this complication. Honig et al. reported advanced endoscopic rescue of a DJ leak after a PPPD in a post-esophagectomy patient with pancreatic cancer [13]. However, the role of endoscopic treatment for this rare complication after PD remains to be established.

Under salvage conditions dismantling the GJ/DJ anastomosis completely, closing the pyloric/gastric and jejunal end, performing a catheter gastrostomy, and feeding jejunostomy may be a bail-out strategy (Fig. 1). Given the rare incidence of GJ/DJ leak after PD, this salvage procedure has been scarcely reported in the literature [14]. However, for esophagogastric resections, it is well described that the anastomosis sometimes has to be taken down in case of fulminant sepsis or large defects. Diversion then needs to be taken into consideration, with restoration of intestinal continuity at a later date [15]. In the two patients in the current study, intestinal continuity could be restored and a GJ was successfully performed approximately 6 months after the dismantling procedure. In the authors’ personal experience, at least 6-month time is necessary to allow healing of the concomitant POPF, resolution of the inflammatory changes, and restitution of nutrition.

## Conclusion

GJ/DJ leak is a rare complication after PD. Prompt diagnosis and early intervention is important, and in most cases, a surgical intervention is necessary for a good outcome. Under salvage conditions, dismantling of the GJ/DJ anastomosis may be necessary. The role of interventional radiological or endoscopic procedures in the management of this complication remains to be established.
